# Forging Unity: European Commission Leadership in the Brexit Negotiations

**DOI:** 10.1111/jcms.13171

**Published:** 2021-01-14

**Authors:** Leonard August Schuette

**Affiliations:** ^1^ Department of Political Science Maastricht University Maastricht

**Keywords:** Brexit, European Commission, EU institutions, leadership, negotiations

## Abstract

This article explains why the European Union has remained strikingly cohesive during the Brexit withdrawal negotiations by focussing on the role played by its negotiator: the European Commission'’s Task Force 50. The analysis demonstrates that the Task Force 50 set out to forge unity among the EU27 by exercising both subtle instrumental and direct political leadership. The Commission significantly influenced the outcome of the negotiations by shaping the agenda and process, brokering deals, and ultimately achieving a withdrawal agreement that all member states signed up to. Its transparent and consultative behaviour generated trust among member states, which allowed the Commission to play such a prominent role. These findings challenge the prevailing view that the EU has become increasingly intergovernmental at expense of the Commission. Drawing on original interviews, the article substantiates this argument by tracing the Commission's leadership activities in the run‐up to and throughout the withdrawal negotiations (2016–20).

## Introduction

The confluence of recent crises has caused almost pathological divisions among the member states of the European Union (EU) (Schuette, 2019; Webber, 2019). The EU was paralyzed and struggled to reach consensus on how to cope with the Euro, Schengen, and rule of law crises. Against this backdrop, Brexit threatened to become the final straw that would lead to the unravelling of the EU (Van Middelaar, 2019, p. 120; Oliver, 2018, p. 256). Then European Commission President Jean‐Claude Juncker (2017) exemplarily dreaded that the ‘Brits will manage without big effort to divide the remaining 27 member‐states’. Contrary to these gloomy predictions, however, the EU remained strikingly united during the Brexit withdrawal negotiations with the United Kingdom (UK) and concluded agreements both with the May and Johnson governments largely on its own terms. While the EU had preferred a closer future relationship than the one transpired, it protected the integrity of the single market, safeguarded the rights of EU citizens in the UK, avoided a hard border in Ireland, and demonstrated the difficulties of leaving the union.

This article seeks to explain this puzzling unity among the EU27 and, by extension, the negotiation success. Extant accounts emphasize asymmetrical bargaining constellations (Schimmelfennig, 2018), negotiation errors on part of the UK (Rogers, 2019), path dependencies (McTague, 2019), the maturing of the EU as a strategic polity (Laffan, 2019), or a combination of rational, bureaucratic, identity, and framing factors (Jensen and Kehlstrup, 2019). This article, instead, specifically zooms in on the role of the EU's actual negotiator: the European Commission in form its Task Force for the Preparation and Conduct of the Negotiations with the United Kingdom under Article 50 (TF50) under the leadership of chief negotiator Michel Barnier. The article traces the role of the TF50 in the run‐up to (June 2016–March 2017) and throughout (March 2017–January 2020) the Brexit negotiations.

It finds that the Commission was acutely aware of the potentially existential threat Brexit posed for the EU and, unlike in other negotiations where the Commission tended to pursue parochial interests, therefore set out to forge unity among member states to protect the overall EU polity. To do so, the Commission exercised both subtle instrumental and direct political leadership which proved crucial in shaping the process and agenda, brokering deals among the EU27, and ultimately achieving the withdrawal agreement that all parties signed up to. The consciously transparent and consultative conduct was critical in producing a trusted and cooperative relationship with the member states and other institutions, which enabled the Commission to show such prominent leadership.

These findings challenge the growing consensus among scholars that the EU has become increasingly intergovernmental (Bickerton *et al*., 2015; Genschel and Jachtenfuchs, 2018). Van Middelaar (2019), for instance, suggests that recent crises posed sensitive questions of redistribution, citizenship and borders, prompting the European heads of states and governments in the European Council to dominate crises responses and circumvent EU institutions and procedures. The Commission's discernible leadership also differs from its roles during the renegotiations of both the UK's membership terms, when it was largely sidelined, and the Transatlantic Trade and Investment Partnership, when it struggled to shape negotiations (Beach and Smeets, 2019; De Bièvre, 2018).

The article advances this argument by, first, discussing theoretical perspectives on leadership in the EU. Second, it outlines the methodological approach. Third, the article assesses the Commission's scope for leadership in terms of institutional, contextual, and personal factors. The fourth part traces the Commission's instrumental and political leadership activities. Finally, the conclusion discusses limitations and offers reflections on the wider applicability of Commission's leadership approach.

## I The Contested Leadership of the Commission

As a fragmented and complex system of multi‐level governance, the EU lacks a singular leadership structure (Toemmel and Verdun, 2017, p. 103). With multiple sources of authority, the EU constitutes an ‘intensely leaderful polity’ (Mueller and Van Esch, 2019, p. 2), where different actors – including the EU institutions, national governments, or non‐governmental actors – can exercise different types of leadership. This article focuses on the most recent typology of leadership as *instrumental* and *political*. Political leadership denotes direct acts of shaping the agenda and building coalitions to reach a consensus on the desired outcome (Ross and Jenson, 2017; Toemmel, 2013), while instrumental leadership refers to institutional activities such as drafting proposals and the use of varieties of expertise to subtly influence an outcome (Beach and Smeets, 2019). It has been the perennial occupation of EU integration theories to offer competing accounts of which (type of) actor possesses the ultimate leadership authority. The classic dichotomous debate between neo‐functionalism and liberal intergovernmentalism on whether supranational institutions or national governments dominate the integration process, however, has recently given way to more nuanced discussions.

New intergovernmentalism (NI) remains in the tradition of liberal intergovernmentalism by identifying the intergovernmental actors in the European Council as the political leaders of the EU. However, it recognizes that delegation of powers to EU institutions have occurred (Bickerton *et al*., 2015). Yet, rather than empowering the traditional supranational institutions such as the Commission, member states have created de novo bodies that tend to be controlled by specific mandates and tight oversight mechanisms. In this new intergovernmental era, there is allegedly little space for Commission leadership. The new institutionalist leadership (NIL) theory shares NI's premise of the centrality of the European Council in post‐Maastricht EU politics, but deviates from NI's conclusions on institutional leadership (Beach and Smeets, 2019; Smeets and Beach, 2020). NIL argues that the current era is characterized by a division of leadership responsibilities between the European Council and the other EU institutions. In the control room, national governments remain in charge of the overall process and provide *political leadership*. The complexity of recent crises, however, required governments to delegate authority to supranational actors in the machine room. In doing so, supranational institutions like the Commission can exert *instrumental leadership* of subtly steering negotiations on EU reforms or with third parties.

With the election of Jean‐Claude Juncker as Commission President in 2014 after the highly politicized *Spitzenkandidaten* process and his declared intention to lead a ‘political Commission’, a third strand of literature on institutional leadership has emerged. This group of scholars is primarily motivated by the conceptual question what ‘political Commission’ designates and the empirical question whether the Juncker Commission indeed constituted such a ‘political Commission’ (Kassim and Laffan 2019; Toemmel, 2019; Nugent and Rhinard,  2019). These scholars rarely explicitly engage with the theoretical debate on institutional leadership and their vantage is hardly a consolidated theoretical approach. However, the findings that the Juncker‐Commission acted unprecedentedly political in substantive (for example the Juncker Plan and White Paper on the Future of Europe), institutional (namely reorganization of the College and presidentialisation of the Commission), and rhetorical terms challenges NIL's premise that the Commission would be confined to providing instrumental, not political, leadership.

These competing perspectives suggests that the interplay of three probabilistic conditions determines what type of leadership, if any, the Commission can exercise: its institutional position in the EU landscape; the context of public and member state opinion vis‐à‐vis European integration; and the personal capacity of potential leaders (Kassim *et al*., 2013; Toemmel, 2013). Those scholars upholding the ‘Commission in decline’ thesis point to unfavourable institutional and contextual developments since the Maastricht Treaty. Institutionally, the continuous empowerment of the European Parliament, the decline of the traditional Community method of policy‐making and concomitant rise of the European Council have come at the expense of the Commission (Puetter and Fabbrini, 2016; Dinan, 2016; Kassim *et al*., 2013; Bauer and Ege, 2012). Contextually, increased politicization of the EU and consequently reluctance among member states to support greater supranational integration allegedly reinforce the unfavourable institutional developments (Keleman and Tarrant, 2011). Empirical studies also note that with the possible exception of Juncker, Commission Presidents since Delors have lacked the personal capacities to exercise significant leadership (Mueller, 2017; Toemmel, 2013, 2019).

These three perspectives also share – to a varying degree – the premise that the relationship between the Commission and other EU institutions is driven by the zero‐sum logic of power maximising institutions. NI and the literature on the ‘political Commission’ assume a power struggle between the Commission and European Council/Council. NIL emphasizes a division of labour, in which, however, the Commission is the subordinate executor of tasks informally delegated by the European Council, even if that allows the Commission to steer proceedings behind the scenes. Yet, recent institutional developments question this hierarchical perspective on the inter‐institutional relationship, and Brexit is a case in point. Not only has the European Council limited administrative resources and sanctioning powers to control the Commission, the parallel process of presidentialisation in the European Council and the Commission – with the creation of the President of the European Council and the incremental empowerment of the office of the President of the Commission – has created a new inter‐institutional dynamic. As a result, the division of labour is often negotiated by the two Presidents, which is conducive to producing more complex and positive‐sum cooperation (Bocquillon and Kassim, 2020).

In sum, this section analysed competing perspectives on the Commission's leadership in order to distil a consolidated theoretical framework based on three probabilistic conditions, which allows formulating expectations on the Commission's leadership and guides empirical assessments beyond this specific case. The framework suggests that the stronger the institutional position of the Commission in the Brexit negotiations, the greater the support among member states for a common approach to the negotiations, and the more astute and prominent the Commission's leadership personnel, the more likely the Commission can exert instrumental and political leadership.

## II Methodological Approach

The ensuing analysis assesses what type of leadership, if any, the Commission exerted, its importance for forging unity of the EU27 during the withdrawal negotiations and, by extension, the successful conclusion of the withdrawal agreements with the May and Johnson governments. While several factors contributed to the negotiation outcome, the limited scope here does not allow for systematically engaging with the aforementioned alternative explanations. Given the emphasis on multi‐causality, the aspiration is to illustrate that the Commission's leadership was one causal factor. Following a congruence analysis of the three theoretical conditions, this paper relies on minimalist process‐tracing to link the Commission's leadership with the outcome, where the causal process is not unpacked into its component parts (Bennett and Checkel, 2014, p. 7). Instead, observable empirical manifestations for each of the leadership strategies need to be made explicit a priori to evaluate their existence and impact.

It adds analytical clarity to distinguish instrumental and political leadership strategies conceptually, even if in practice they often overlap. Instrumental leadership strategies consist of drafting legal texts and position papers as well as utilizing expertise to influence the outcome. By holding the pen, the Commission can construct the scaffolding for subsequent debate (Beach, 2004), while its expertise on policy, procedure, legality and political context allows it to frame the debate in terms favourable to its goals, for instance by presenting policy options without alternatives or influence the perception of payoffs of the different options (Barnett and Finnemore, 2004). Observable indicators for the provision of expertise include position papers and information sheets, hosting of technical seminars, and organization of the TF50 to effectively draw on the wider expertise of the Commission. Drafting is observable through textual evidence of draft position papers and negotiation texts.

Political leadership strategies, in turn, refer to agenda shaping and brokerage activities. By raising awareness and active framing (agenda‐setting), prioritising items on the agenda (agenda‐structuring), and wilfully circumventing other matters (agenda‐exclusion), the Commission can shape the negotiation agenda in its favour (Tallberg, 2003). By building coalitions with an array of similar‐minded actors and sounding out member‐states' concerns to identify, and subsequently push for, possible zones of agreement, the Commission can also act as central compromise broker among member‐states. Empirical footprints for agenda‐shaping include the mandate and negotiation directives, media interaction, the communication of red lines, progress reports, draft agreements, and interviews with negotiators. Evidence for brokerage, in turn, can be found in shuttle diplomacy by the negotiator and bilateral meetings with stakeholders, coalition‐building through intensified interaction with a particular set of actors, drafting of single negotiation texts, and interviews with involved negotiators.

Fourteen semi‐structured original interviews with EU officials from the TF50, European Parliament's Brexit steering group, the European Council, as well as national officials directly involved in the negotiations (that is, Brexit delegates) from a diverse range of member states such as Germany, Italy, and Poland serve as the primary source of data. The interview response rate was approximately 60 per cent. Publicly available information such as official documents published by the TF50, press statements, media interviews, and journalistic accounts were used to triangulate the evidence generated in the interviews.

## III The Commission's Scope for Providing Leadership in the Withdrawal Negotiations

This section evaluates the presence in the withdrawal negotiations of the three conditions identified in the theory section that explain the exercise of leadership: the Commission's institutional position in the negotiations, the context of member states' sentiments and interests in the withdrawal negotiations, and the qualities of its leadership duo.

### Institutional Position, the TF50's Set‐up and the Appointment of Michel Barnier

Due to the unprecedented nature of Brexit, no institutional blueprint existed how the EU would conduct the looming negotiations with the UK. The EU had to devise new structures and *modi operandi*, which eventually produced the institutional set‐up that enabled the Commission's leadership. By December 2016, the EU had translated the vague Article 50 stipulations into a multifaceted governance system for the Brexit negotiations (European Council, 2016). The European Council would sit at the helm of the negotiations with the powers to set the negotiation guidelines, determine whether ‘sufficient progress’ had taken place on the first phase issues, and grant extensions to the withdrawal period. The Commission's TF50 was appointed lead negotiator, which endowed it with greater bargaining power than during reform negotiations or crisis management, when EU member states tend to circumvent existing procedures in which EU institutions possess an important formal role. The exclusive conduct of negotiations via the TF50 put it into a commanding position of influence (Gostyńska‐Jakubowska and von Ondarza 2020). The General Affairs Council (GAC) would coordinate the withdrawal process with the Commission. It thereby also served as a control organ to ensure that Commission abided by the negotiation guidelines. Member states appointed Belgium diplomat Didier Seeuws as the chair of the GAC's Working Party on Article 50. Last, the European Parliament set up a Brexit Steering Group under the chairmanship of Guy Verhofstadt.

In line with Juncker's wider ambitions to deploy the Commission's expertise for political priorities, he chose a distinct set‐up of its negotiation force. While negotiations teams usually only consist of Commission officials, Juncker superimposed a political figure on this technocratic structure: Michel Barnier. Juncker and his team had feared that the Leave side would win the referendum and made contingency plans prior to 23 June. Aware of the constitutional rather than technical nature of the looming negotiations, Martin Selmayr, then Juncker's chief of staff, suggested to approach Barnier rather than Jean‐Luc Demarty, as the Director General for Trade in the Commission the natural choice in normal trade negotiations (Interview #13). When formally appointing him head of the TF50 on 1 October 2016 Juncker duly stressed Barnier's political credentials: ‘I wanted an experienced politician […] He has an extensive network of contacts in the capitals of all EU Member States and in the European Parliament’ (Juncker, 2016). A former French cabinet minister and two times European Commissioner, including for the internal market, Barnier possessed deep knowledge of national and EU politics as well as relevant technical matters, and was thus in a unique position to transcend the political‐technical divide. The appointment of a well‐connected and experienced political heavyweight endowed the TF50 with greater authority and proved critical in persuading national leaders to step back and grant the formal negotiation mandate to the TF50 in December 2016 (Interviews #6; #11; McTague, 2019).

Barnier had at his disposal some of the most experienced and brightest officials from the Commission. Sabine Weyand, who was as Deputy Director‐General of DG Trade closely involved in the trade negotiations with Canada (CETA) and the United States (TTIP), was appointed his deputy. At its peak in the summer of 2018, the TF50 had almost 60 permanent members of staff. Furthermore, each TF50 staff member had a point of contact in one of the Commission's DGs and thus access to the entire firepower of the 33,000 people strong Commission bureaucracy (Interview #6).

Thus, the Commission was in the institutionally powerful position of lead negotiator. Yet, unlike in normal trade negotiations the European Council and GAC were more closely involved, implying little room for manoeuvre for the Commission to escape from its mandate.

### Interest Constellation of the EU27: Polity Interests

The context within which the withdrawal negotiations took place differed from previous episodes when rising Euroscepticism sentiments across the continent had undermined the scope for Commission leadership. Following the shock of the Brexit vote, which was to be aggravated by the election of Donald Trump as US President in November 2016, member states were more willing to put existing differences aside. Moreover, they were generally uncertain about the procedure and substance of the negotiations, which opened the door for the Commission to proactively shape key early decisions, including its appointment as lead negotiator.

Above all, the prerequisite for the trusted and cooperative inter‐institutional relationship that enabled the Commission's leadership was a particular interest constellation. Unlike in ordinary trade and reform negotiations, where the Commission would be expected to have distinct policy and bureaucratic interests, in the withdrawal negotiations the Commission primarily had *polity interests*. Brexit posed a peril to the very survival of the Union: it threatened not only to unleash a chain reaction by setting a precedent for other states to leave, but also the integrity of the single market should the UK be allowed to opt‐into parts of it, and the pacification of the island of Ireland underwritten by the EU. The Commission recognized the momentous nature of the withdrawal negotiations early on; maintaining cohesion became not only a means to a better negotiation outcome but an end itself to preserve the Union (Interviews #1, #6, #13). The TF50 had no interest to deviate from the negotiation mandate to pursue parochial gains and risk both undermining trust as well as incentivising individual member‐states to break with the common line.

At the beginning of the negotiations, however, it was far from clear that the member states would share the TF50's outlook. Most recognized the importance of the integrity of the single market and shared broad interests on the three concrete issues of the first phase of the negotiations (Kassim and Usherwood, 2017; Interviews #2, #5, #9). However, member states differed substantially in their economic exposure and political ties to the UK, creating varying incentives to stray from the common negotiation position and make concessions to the UK (for example Chopin and Lequesne, 2020). Due to deeply intertwined political histories and economic relations, Ireland was more affected than any other member state (Laffan, 2017). EU membership provided the crucial context, in which Anglo‐Irish relations improved and normalised. The single market rendered the border between the Republic of Ireland and Northern Ireland de facto invisible in daily life and the EU played an important role in underwriting peace on the island. For some other member states such as the Netherlands, Denmark, or Poland, the UK was one of the most important trading partners. Meanwhile, Germany and the Nordic states considered the UK a crucial political ally in advancing a liberal economic agenda and providing teeth to the EU's foreign policy. For Central and Eastern European states, the UK was crucial in balancing the Franco‐German couple and curtailing supranational tendencies (Turner *et al*., 2018). Member states thus had to balance particularistic interests vis‐à‐vis the UK with wider polity interests (Laffan, 2019).

It is therefore simplistic to claim with hindsight that the EU's unity was a predestined conclusion (Jensen and Kehlstrup, 2019, p. 2; Glencross, 2018, p.188). Among national and EU officials alike, there was a distinct fear at the onset of the negotiations that the UK would successfully divide‐and‐conquer the member states (Interviews #2, #5, #6, #7). Moreover, it was far from obvious that the other member states would risk negotiation priorities such as maximising the financial settlement by protecting the idiosyncratic interests of a small country like Ireland (Laffan, 2019, p. 10). Hence, the interest constellation prior to the negotiations was not determinate. While there were some key homogeneous interests, member states priorities varied. It arguably needed the Commission to mould the different priorities into a common position all member states could support.

In sum, the Commission found itself in an institutionally powerful position as lead negotiator, though with the member states closely involved; operated in an environment of uncertainty among member states and indeterminate interests constellation that was potentially conducive to allowing the Commission a greater role than hitherto; and its Brexit efforts were led by the prominent duo of Juncker and Barnier. This interplay of institutional, contextual and personal factors was conducive to the Commission exercising instrumental leadership, but due to the close involvement of the member states it was unclear whether it could exercise political leadership. As such, the withdrawal negotiations differed from previous negotiations, where the Commission exercised only modest influence. By way of example, the Commission had no formal role in the UK renegotiations of its membership terms prior to the referendum, which was led by then‐President of the European Council Donald Tusk. Its taskforce was headed by a Commission official – Jonathan Faull – rather than a political figure and the negotiations took place before the twin shocks of Brexit and the election of Donald Trump that threatened the integrity of the EU and the international rules‐based order respectively (Beach and Smeets, 2019; Eckert, 2018).

## IV The Commission's Leadership in during the Withdrawal Negotiations

The following section examines the empirical evidence of the withdrawal proceedings to assess whether the Commission succeeded in exploiting this constellation of institutional, contextual, and personal factors. The ensuing sections flesh out the components of both instrumental and political leadership strategies and evaluates their significance in precipitating the negotiation outcome. 1

### Instrumental Leadership: Subtly Steering the Negotiations

The immense technical complexity of the withdrawal negotiations produced a strong demand for instrumental leadership by the Commission. Indeed, the TF50 used its technical and legal expertise as well as drafting skills to delineate the contours of the negotiations. Once the TF50 was set‐up in the autumn of 2016, it engaged in a massive exercise of reviewing the *acquis communautaire* – the entire body of EU law – to map the implications of the variants of Brexit for different EU policy fields (Laffan, 2019, p. 9). Aided by the Commission's DGs, legal service, and Council Secretariat, the members of the TF50 unraveled the extreme complexity of Brexit to identify the consequences of the UK's emerging red lines (Interviews #1, #6). Diplomatic meetings on the technical level in Brussels between national delegations, the Council's Task Force, and members of the TF50 in parallel to Barnier's shuttle diplomacy (see below) facilitated early consultations.

The TF50's early demonstration that it had a comprehensive grasp of the Brexit technicalities and its extensive consultations with central stakeholders proved critical in persuading those member states sceptical of the Commission leadership, such as Poland, or with significant stakes in the negotiations, such as Ireland, to rally behind the TF50 (Interview #8). When Michel Barnier and some of his senior staff met an Irish delegation on 12 October 2016, the latter were struck by his already existing understanding of the Good Friday Agreement and the potentially critical consequences Brexit entailed for peace on the island of Ireland (Connelly, 2018, p. 67). The TF50 had been aware of the land border issues not only in Ireland but also pertaining to Gibraltar and Cyprus from the early autumn of 2016 (Interview #1). In a recognition of the technical complexity and nascent political importance of the Irish border issue, in mid‐2017 the TF50 assigned Nina Obermaier, a former official at the EU's diplomatic service where she handled the complex EU‐Swiss relations, to deal with the Irish dossier (Interview #6). Given its historic links to the UK, the Irish government had been tempted to engage with London bilaterally. However, it realised in in late 2016 that London lacked the political will and means to provide solutions to the border issue, and unequivocally backed the TF50, which was epitomised by its rejection of calls for a bilateral deal by the House of Lords in December 2016 (House of Lords, 2016; Connelly, 2018, p. 79ff; Interview #1).

By the time Theresa May triggered Article 50 in March 2017, the TF50 had cemented its role as sole negotiator and built up deep expertise across several affected policy dossiers, which would serve not only as a source of a permanent dominance by the TF50 over its UK counterparts, but also allow it to shape the tracks along which the negotiations would proceed. As one observer noted, the ‘TF50 was patently on top of its brief’ (Green, 2017). Indeed, the TF50 was quick off the starting blocks to gain an early mover advantage over the UK. Over the course of the first three months of negotiations alone, it published 14 position papers on withdrawal issues ranging from the financial settlement to data protection (European Commission, 2020). From the start, the negotiations thus took place on the TF50's terms (interview #5).

The Irish border issue soon emerged as the politically thorniest and technically most challenging issue among the three main baskets. Throughout the referendum campaign, the Irish border issue had barely featured in public debates in the UK. This apparent lack of serious engagement continued in the early months of the negotiations, when the UK demonstrated a striking ignorance of the technical and legal challenges Brexit posed to North–South cooperation and the Good Friday Agreement more broadly, which was reflected in two position paper published on 16 August that vaguely referred to innovative technological solutions (UK Government, 2017).

In response, the TF50 in close collaboration with Irish officials drew on its expertise to arrive at a creative solution. On 9 November 2017, an internal paper was leaked, in which the TF50 argued that there should be no ‘regulatory divergence’ by Northern Ireland from the rules of the EU's single market and the customs union – this was the genesis of the ‘backstop’ that was to shape the negotiations until its very conclusion (Telegraph, 2017). The ‘backstop’ proved the crucial door opener for the next phase of negotiations. Despite outcries on the Conservative backbenches, in the British press, and from the Democratic Unionist Party in Belfast, UK and EU negotiators agreed on a joint report on 8 December 2017 with the backstop as its centrepiece. It was obvious that the situation in Ireland would remain a protracted political problem in the UK, but the TF50 together with Irish diplomats engineered a creative compromise solution. Article 49 of the joint report offers three solutions to avoid a hard border: (1) a trade deal, implying that the UK would stay in the single market and customs union; (2) specific solutions proposed by the UK; and (3) ‘in the absence of agreed solutions’, the ‘backstop’. Notwithstanding that the first two options appeared unrealistic from the start, the wording allowed Theresa May to sign up the joint report, after the Democratic Unionist Party had threatened to end its confidence‐and‐supply arrangement with the Conservative Party. The December compromise thus managed to resolve the issue by delegating the border issue to the next phase, yet with the critical caveat that recourse to the backstop would be the default option. It was thus the TF50's internal paper, paired with subsequent creativity, that laid out the tracks for the joint report (Connelly, 2018, p. 348ff.).

In light of the looming deadline to finalise the Withdrawal Agreement in October 2018, the next episode in which the TF50 effectively used its expertise and drafting position to shape the outcome of the negotiations emerged soon after the joint report. In mid‐January 2018, the TF50 set out to translate the political agreement into a legal text and draft the first version of the Withdrawal Agreement. Before the draft was circulated to the UK and published on 28 February, the TF50 sent versions to all national delegations. Once again drawing on its immense expertise, the TF50 received, answered, and in part incorporated into the draft 700 questions from member‐states over the course of a few days ensuring a widespread sense of collective ownership (Interview #1; Laffan, 2019, p. 9). This pace and level of consultation not only further increased member states' trust in the TF50 (Interview #11). The timely publication also again allowed the TF50 to lay out the tracks as the ensuing negotiations would be based on the EU's document (Green, 2018). It included the legal operationalisation of the backstop in form of a ‘common regulatory area’ between the EU and Northern Ireland, not the entire UK as Theresa May wanted (European Commission, 2018: Art. 3).

The backstop was to remain a sticking point with negotiators agreeing on an UK‐wide backstop in the November 2018 Protocol on Ireland, only for Boris Johnson to agree for Northern Ireland to remain aligned to EU rules to avoid a hard border, in what closely resembled the EU's draft Withdrawal Agreement. In sum and as expected by the NIL perspective, the TF50 utilized its deep subject knowledge and drafting skills to gain negotiation advantages over the UK, pave the way of the negotiation, and generate trust among member‐states. The TF50 discernible exercise of instrumental leadership thus contributed to the unity of the EU27.

### Political Leadership: Shaping Key Decisions of the Negotiation

The previous section demonstrated that the TF50 subtly shaped the negotiations by using its expertise and drafting skills. The following section shows that despite the close involvement of the member states, it also exercised more direct forms of political leadership in form of agenda‐shaping and brokerage, which was accepted by the member states and the GAC working party who trusted the Commission not to pursue parochial gains. While President of the European Council Donald Tusk was a prominent and vocal public persona, he never undermined the TF50. Significantly, all political decisions throughout the negotiations by the TF50 were taken in close coordination with President Juncker and his cabinet – not the wider College of Commissioners given the Presence of the UK European Commissioner Julian King – which added both weight and legitimacy to its handlings (Interview #13).

The most strategic and consequential episode of agenda‐shaping by the TF50 occurred at the beginning of the process. Following the unexpected referendum result, most member states were uncertain about their interests in the forthcoming negotiations. While the leaders had laid out broad principles in an informal meeting on 29 June 2016, few member states had expected and adequately prepared for the Vote Leave side to win the referendum (Interview #11). There was a vacuum and the TF50 proactively moved to fill it. The TF50 used its aforementioned technical grasp to design the overall framework of the negotiations. First, it identified and then put three major baskets as priorities on the agenda – citizens' rights, financial settlements, and the Irish border issue – holding technical seminars on those matters with all member states to get them on board. Second, in private talks with key member states in January 2017, Barnier and his senior staff presented the idea to divide the negotiations into two distinct phases: the first phase would address withdrawal matters, while the second phase would focus on future relations (Interview #1, #9, #11). Only if ‘sufficient progress’ on withdrawal matters had been reached would the negotiations proceed to the next phase.

Described by one national official as a ‘stroke of genius’ on part of the Commission (interview #11), this example of agenda‐structuring would allow the EU to control the process: only once the UK had settled its bills and found a solution to avoid a hard border in Ireland would talks about a future trade deal commence (McTague, 2019; Rogers, 2019). Due to the asymmetrical interdependence between the EU and the UK – in 2015, the exports to the EU accounted for 44 per cent of UK exports compared to less than 7 per cent vice versa (ONS, Office for National Statistics, 2016) – the UK needed a trade deal much more than the EU, thus depriving the UK much of its leverage (Schimmelfennig, 2018). The TF50 also temporarily excluded potentially divisive issues among the EU member states regarding future trade relations from the agenda, thereby strengthening unity on withdrawal matters. Convinced by the merits of the proposal, the national leaders duly included the phased approached in the negotiation mandate of 29 April 2017. Phasing the negotiations was no legal necessity – there is no reference to it in Article 50 – but an astute political choice devised by the TF50 that significantly strengthened the EU's hand.

In addition to this agenda‐shaping, the TF50 alongside other EU actors also effectively framed the negotiations to shape the influence narratives and perceptions of the negotiations with the objective to highlight the difficulties involved in exiting the EU to bolster support for the EU. By publishing draft agreements, guidelines, negotiation directives, position papers, and agendas for negotiation rounds, the TF50 used transparency as a negotiation tool (Council of the European Union, 2017). It successfully created the impression that it was both united and well‐prepared for the negotiations, in stark contrast to an opaque and divided UK government (Jensen and Kehlstrup, 2019; Rogers, 2019). Indeed, this strategy seems to have been successful as Eurosceptic parties across the continent no longer want to leave the union and support among the public for membership across the EU increased from 53 per cent in September–October 2016 to 62 per cent in September 2018 (Chopin and Lequesne, 2020; De Vries, 2017; Eurobarometer, 2018). Suave, serious, and well‐known, Michel Barnier played a crucial role in the EU's framing efforts. As the public face of the EU, his remarks on the Brexit negotiations at press conferences following negotiation rounds or rare interviews resonated widely. Especially his repeated warning that ‘the clock is ticking’ struck a chord with European publics, even becoming the title of an ARTE documentary (Laffan, 2019). It appears implausible that a technocrat from the Commission could have played a similarly effective public role.

The TF50, furthermore, became the central broker of compromises among the EU27. The Barnier Method comprised three levels, on which extensive coordination and consultation took place to sound out the concerns among member states (Interview #14). First, Barnier himself travelled widely, visiting every capital at least twice, and liaised with national parliamentarians, businesses, trade unions, and local citizens, symbolically visiting the Irish border and Danish harbours. He thereby countered the narrative of aloof technocrats in Brussels and ensured collective ownership of the Withdrawal Agreement among stakeholders across the continent (Interview #7). Second, the Commission engaged bilaterally with member states on an informal level, hosting more than 150 meetings with national delegations and regular pre‐ and debriefs before and after negotiation rounds (Interview #1). Several national officials emphasized the unprecedented transparency of the Commission's conduct in stark contrast to previous trade negotiations (Interviews #5, #8, #9). One early example of the high‐level diplomatic exchanges was Juncker's successful effort in two private meetings in August and September 2017 to convince the reluctant German chancellor Merkel to keep the Irish border as an issue to be included in the Withdrawal Agreement (Interview #13). The German government had repeatedly questioned the need to deal with the border in the first phase of the negotiations, referring to its potential to derail the negotiations.

Third, the TF50 forged synergetic relations with the other EU institutions. It co‐operated closely with the Council's working party under Didier Seeuws, with senior TF50 staff attending working party meetings three times a week during the course of the negotiations. Aware of the need for its consent at the end of the process, the TF50 also fully involved the European Parliament in the negotiations by granting it extensive level of information and access to the negotiation process (Interview #4). Barnier regularly attended and updated the European Parliament's Brexit steering group to ensure inter‐institutional unity (Closa, 2019). The TF50 exploited the cohesion among the EU institutions to strengthen its negotiation position vis‐à‐vis the UK by using the European Parliament's red lines on particularly citizens' rights, for instance on family reunions and applications for settled status, to push for a more ambitious agreement (Bressanelli *et al*., 2019). Thus, the Commission's conduct allowed for unprecedentedly close inter‐institutional cooperation, which enabled it to exercise political leadership unopposed by the member states (Kassim, forthcoming).

The resulting status as trusted broker proved critical in reaching the final deals with both the May and Johnson government. When the negotiators agreed to the Withdrawal Agreement on 13 November 2018, the TF50 took the initiative to reach a deal by conceding that the backstop would entail an all‐UK rather than Northern Ireland‐only customs union, which to many member states came as an unexpected and concerning development and prompted level‐playing field concerns (Interviews #12, #14; O'Rourke, 2019, p. 281). Between the 13 November and the European Council on 25 November, TF50 negotiators managed to assure member‐states that the EU would maintain its leverage in future trade negotiations and that the level playing fields provisions including in the deal were watertight (Interview #1, #9). However, as one official noted, ‘in normal circumstances, this would not have gone through’ in such a short span of time (Interview #12).

In a similar vein, the TF50 could rely on its reputation when it convinced member‐states to endorse the revised Withdrawal Agreement on 17 October 2019. Negotiations between the TF50 and its UK counterparts on a revised Protocol on Ireland and Northern Ireland dragged on deep into the night of 16 October, with the final text only being circulated among member‐states at noon on 17 October, three hours before the European Council began. In light of the time pressure of the looming Brexit deadline on 31 October, member‐states trusted TF50's reassurances that the deal reflected its interests (Interview #2).

In sum, the Commission actively shaped the agenda of the withdrawal negotiations and was critical in brokering compromises among the member states to reach a final deal. Contrary to the expectations in the literature, the inter‐institutional relationship was characterized by extensive consultations, transparency, and ultimately trust, which led member states to welcome the Commission's political leadership.

## Conclusion

This article has investigated the causes for the puzzling unity among the EU27 during the Brexit withdrawal negotiations by tracing the role played by the Commission's TF50. The empirical findings plausibly affirm that alongside other causal factors, indeed, the TF50 contributed significantly to the successful negotiation outcome. In contrast to the preceding renegotiations of the UK's membership terms, the Commission was in an institutionally powerful position, operated in a context of uncertainty and shock among member states, and was led by the effective leadership couple of Juncker and Barnier. The TF50 transparent and consultative conduct in the pursuit of its policy interest generated trust among the member states, which therefore accepted its instrumental and political leadership. It provided legal and technical expertise, drafted key documents, shaped the agenda, and brokered compromises. Backed by Juncker behind the scenes, Michel Barnier's role not only as chief negotiator but also public face of the EU during the negotiations and shuttle diplomat proved crucial in granting the TF50 authority in the eyes of the member‐states and the public.

These findings make two central contributions to the existing literature. First, the article nuances the debates on inter‐institutional dynamics in the EU. It lends evidence to the claim that the Commission generally and the Juncker Commission specifically can exert political leadership. Indeed, the Commission's role in the withdrawal proceedings proved more influential than the intergovernmental narrative suggests and more political than the new institutionalist leadership perspective expects. It is undoubtable that at a time when the EU increasingly affects core state powers, member‐states will seek to be closely involved in the decision‐making processes, which is corroborated by their active involvement in the withdrawal negotiations. However, this does not inevitably mean a shift of power from institutions to national governments and the concomitant decline of the Commission, but may give rise to a more complex, positive sum collaboration among the EU actors.

Second, it identifies conditions under which the Commission can exert political leadership, which is hitherto underspecified in NIL and literature on the ‘political Commission’. The analysis demonstrated that favourable institutional and contextual factors were necessary but insufficient for the political leadership by the Commission. It required the conscious efforts by the Commission's leadership couple of Juncker and Barnier to exploit this favourable constellation by building symbiotic relationships with other EU institutions, which allowed the Commission to exert political leadership to forge unity. This inclusive approach stands in marked contrast to previous episodes when the Juncker Commission sought to assert leadership insensitive to the concerns of key member states and other institutions – Juncker's failed intervention during the bail‐out negotiations with Greece or the Commission's proposed refugee relocation quotas are cases in point (see Toemmel, 2019).

Brexit is, of course, an exceptional case. Time will tell whether the context‐specific insights on the agential qualities of the Commission possess external validity (see Debre and Dijkstra, 2020 forthcoming; Schuette, 2020). The negotiations on the future relations that nominally started in March 2020 will provide the first test case for the continued cogency of the Commission's leadership. Some contextual factors differ, as interests among the EU27 are more heterogeneous on economic and security cooperation with the UK than on withdrawal matters and national parliaments will likely have to ratify the deal, both of which will make it more challenging for the TF50 to maintain unity. Beyond Brexit, the Commission's leadership may also provide a governance template for other delicate policy fields such as major trade negotiations or EU foreign policy. Here, too, the EU could conceivably benefit from appointing an actor with a clear political mandate who, sensitive to the interests of the member states and the European Parliament, can draw on the existing technocratic structures to exercise leadership on behalf of the EU.

## Disclosure Statement

No potential conflict of interest was reported by the authors.

## Author Biography

Leonard August Schuette is a doctoral researcher at Maastricht University. Previously, he was a research fellow at the Centre for European Reform (CER), a think tank in London. He holds an MPhil in International Relations and Politics from the University of Cambridge.

## Funding Information

This article is part of a project that has received funding from the European Research Council (ERC) under the European Union's Horizon 2020 research and innovation programme (grant agreement No 802568).

## Interview references

(1) EU official, 26 November 2019, Brussels.

(2) National official, 3 December 2019, Brussels.

(3) National official, 3 December 2019, Brussels.

(4) EU official, 3 December 2019, Brussels.

(5) National official, 13 December 2019, Videocall.

(6) EU official, 18 December 2019, Brussels.

(7) National official, 18 December 2019, Brussels.

(8) National official, 18 December 2019, Brussels.

(9) National official, 13 January 2020, Videocall.

(10) National official, 6 March 2020, Brussels.

(11) National official, 6 March 2020, Brussels.

(12) EU official, 8 April 2020, Videocall.

(13) EU official, 16 April 2020, Videocall.

(14) EU official, 27 April 2020, Videocall.

## Supporting information


**Data S1.** Appendix: Overview of the key moments in the Brexit withdrawal negotiations

## Footnotes

^1^

The Online Appendix offers a timeline and description of the key moments of the withdrawal negotiations.
