# Supplementary material for: Forging Unity: European Commission Leadership in the Brexit Negotiations
Source: J Common Mark Stud. 2021 Jan 14;59(5):1142–59. doi: 10.1111/jcms.13171 (PMC8597162; doi:10.1111/jcms.13171)
Supplement: Supplementary file 1 — Data S1. Appendix: Overview of the key moments in the Brexit withdrawal negotiations [file JCMS-59-1142-s001.docx]

**Appendix: Overview of the key moments in the Brexit withdrawal negotiations**

| **Date** | **Event** |
| --- | --- |
| 23 June 2016 | UK public votes to leave the EU |
| 24 June 2016 | Statement by four EU presidents setting out key priorities |
| 28-9 June 2016 | European Council affirms ‘no negotiation before notification’ |
| 13 July 2016 | Theresa May becomes UK prime minister |
| 27 July 2016 | Juncker appoints Michel Barnier as chief negotiator |
| 2 October 2016 | May confirms that UK will trigger Article 50 by the end of March 2017 |
| 15 December 2016 | European Council confirms the procedure and institutional governance of Brexit negotiations |
| 17 January 2017 | May delivers Lancaster House speech, setting out red lines |
| 29 March 2017 | May triggers Article 50 to leave the EU |
| 31 March 2017 | European Council publishes draft negotiation guidelines |
| 29 April 2017 | Special European Council adopts negotiation guidelines |
| 3 May 2017 | Commission publishes draft negotiation directives on the priorities for the first phase of negotiations |
| 22 May 2017 | European Council adopts negotiation directives and authorises opening of negotiations with UK |
| 8 June 2017 | Snap elections produce hung parliament in UK |
| 19 June 2017 | Formal negotiations between the EU and the UK begin |
| 16 August 2017 | UK publishes position paper on Irish border issue |
| 7 September 2017 | Commission publishes guiding principles on Ireland and Northern Ireland |
| 22 September 2017 | May delivers Florence speech, signaling agreement on financial settlement |
| 20 October 2017 | European Council does not confirm sufficient progress |
| 4 December 2017 | May meets Juncker in Brussels, failing to reach agreement after intervention from DUP |
| 8 December 2017 | Commission recommends that sufficient progress has been made following publication of joint EU-UK report |
| 15 December 2017 | European Council agrees that sufficient progress has been achieved in the first phase of negotiations to move to second phase |
| 20 December 2017 | Commission recommends draft negotiating directives for second phase |
| 29 January 2018 | General Affairs Council adopts negotiation directives for second phase |
| 28 February 2018 | Commission publishes draft Withdrawal Agreement translating into legal terms the joint report |
| 19 March 2018 | EU and UK negotiators update draft Withdrawal Agreement |
| 23 March 2018 | European Council adopts guidelines on the future relationship |
| 19 June 2018 | EU and UK negotiations publish Joint Statement outlining further progress in negotiations |
| 29 June 2018 | European Council highlight that no substantial progress had been achieved on agreeing a backstop solution for the Irish border issue |
| 6 July 2018 | UK government publishes Chequers plan |
| 19-20 September 2018 | Informal European Council in Salzburg rejects Chequers plan |
| 17 October 2018 | European Council notes that not enough progress has been achieved to conclude final agreement with the UK |
| 14 November 2018 | Commission and UK negotiators agree on Protocol on Ireland and Northern Ireland; Commission recommends that decisive progress has been made allowing conclusion of final Withdrawal Agreement |
| 25 November 2018 | European Council endorses the draft Withdrawal Agreement |
| 15 January 2019 | Withdrawal Agreement rejected in the House of Commons |
| 7 February 2019 | May meets Juncker and Tusk, who state that Withdrawal Agreement cannot be reopened but modify language in Political Declaration |
| 11 March 2019 | May and Juncker agree on clarifications relating to backstop and Political Declaration |
| 12 March 2019 | Withdrawal Agreement rejected for second time in House of Commons |
| 20 March 2019 | UK government requests extension of the Article 50 period beyond 29 March 2019 |
| 21 March 2019 | European Council agrees to extension to 22 May 2019 or 12 April 2019 depending on outcome of vote in House of Commons |
| 29 March 2019 | Withdrawal Agreement rejected for third time in House of Commons |
| 5 April 2019 | UK governments requests further extension to 30 June 2019 |
| 10 April 2019 | European Council grant extension until 31 October 2019 |
| 23-6 May 2019 | UK participates in European Parliament elections |
| 24 July 2019 | Boris Johnson becomes UK prime minister |
| 17 October 2019 | Commission recommends endorsing revised Protocol on Ireland and Northern Ireland and Political Declaration; European Council endorses revised deal |
| 19 October 2019 | UK governments requests extension until 31 January 2020 |
| 29 October 2019 | European Council extends Brexit deadline until 31 January 2020 |
| 12 December 2019 | Snap elections in UK produce Conservative majority |
| 29 January 2020 | UK ratifies Withdrawal Agreement |
| 30 January 2020 | EU ratifies Withdrawal Agreement |
| 31 January 2020 | UK leaves the EU and transition period begins |

***The four stages of the Brexit negotiations***

The withdrawal negotiations *de facto* comprised four distinct stages. The pre-notification stage lasted from the Brexit referendum in the UK on 23 June 2016 to 29 March 2017. Negotiations between the EU and the UK had not formally begun, and the EU used that time to prepare for the negotiations. It set up the TF50 and agreed upon an overall Brexit governance.

The negotiations with the UK commenced when Theresa May triggered Article 50 on 29 March 2017, marking the beginning of the second stage (phase 1 of the negotiations). The EU swiftly agreed upon its negotiation guidelines, therein adopting a ‘two-phased approach’. In the first phase, matters relating to the withdrawal would have to be dealt with (EU citizens’ rights in the UK, financial settlement, Irish border). Only if sufficient progress had been achieved on these matters would the EU move on to the second phase. The Irish border proved the thorniest issue and negotiations were only unlocked when the two sides agreed on an insurance mechanisms (backstop), whereby Northern Ireland would remain aligned with the rules of the EU’s single market and customs union if no alternative solution could be found. On 15 December 2017 the European Council decided that ‘sufficient progress’ had been achieved.

The ensuing third stage (phase 2 of the negotiations) was dedicated to finalising withdrawal matters, transitional arrangements, and the future relationship. The UK government published its Chequers plan on 6 July 2018, in which it, among others, proposed to remain in the EU’s single market for goods and to create a combined EU-UK customs territory to render obsolete border controls in Ireland. At an informal European Council in Salzburg on 19-20 September 2018, the EU rejected both proposals. On 14 November 2018, the EU and UK negotiators agreed on a Protocol on Ireland and Northern Ireland, paving the way for an endorsement by the European Council of the final the Withdrawal Agreement on 25 November 2018.

The last stage entailed the ratification of the Withdrawal Agreement in the UK parliament. Following three rejections of the deal in the House of Commons and Theresa May’s resignation, the EU extended the Brexit deadline. Boris Johnson became UK prime minister in July 2019 and set out to renegotiate the deal. On 17 October, the EU endorsed a revised Withdrawal Agreement and Political Declaration. The ‘backstop’ was replaced by a ‘frontstop’, whereby Northern Ireland remained aligned to the EU’s single market and customs union. The Brexit deadline was again extended to 31 January 2020 to allow for a general election in the UK. With a Conservative majority in the House of Commons, the UK left the EU on 31 January 2020.
